# Preparation and Safety Evaluation of Topical Simvastatin Loaded NLCs for Vitiligo

**DOI:** 10.34172/apb.2021.011

**Published:** 2020-11-07

**Authors:** Sahar Yazdani Ashtiani, Saman Ahmad Nasrollahi, Atefeh Naeimifar, Aubid Nassiri Kashani, Aniseh Samadi, Somayeh Yadangi, Ehsan Aboutaleb, Poorya Abdolmaleki, Rassoul Dinarvand, Alireza Firooz

**Affiliations:** ^1^Pharmaceutical, Cosmeceutical and Hygienic Formulation Lab, Center for Research & Training in Skin Diseases & Leprosy, Tehran University of Medical Sciences, Tehran, Iran.; ^2^Department of Pharmaceutics, School of Pharmacy, Tehran University of Medical Sciences, Tehran, Iran.; ^3^Department of Pharmaceutics, School of Pharmacy, Guilan University of Medical Sciences, Rasht, Iran.

**Keywords:** Nano lipid carriers, Probe sonication, Simvastatin, Safety, Vitiligo

## Abstract

***Purpose:*** Vitiligo is a long-term common autoimmune disease in which growing patches of skin lose their color. There is no FDA-approved treatment for vitiligo. However, recent studies have demonstrated an immunosuppressive effect on vitiligo lesions in mouse models by simvastatin. A topical formulation was prepared containing simvastatin-loaded nano lipid carriers (simNLCs) for vitiligo treatment followed by evaluating their physicochemical characteristics and clinical safety.

***Methods:*** Both the lipid phase and the aqueous phase were heated to 75°C separately, and then simvastatin was dispersed in the lipid phase added to the aqueous phase. The mixture was homogenized for 1 minute, then for Nanostructured Lipid Carriers (NLC) formation, the emulsion was sonicated using a probe sonicator. The simNLCs produced were evaluated for drug entrapment, particle size and morphology, zeta potential, polydispersity index, viscosity, drug content, in vitro drug release, in vivo skin safety test, and long-term stability studies.

***Results:*** Dynamic light scattering, transmission electron microscopy and differential scanning calorimetry techniques proved the formation of a stable formulation containing spherical particles with nanoscale size. The drug entrapment efficiency and the drug-loading capacity were determined to be 99.27% and 3.9%, respectively. Human safety results indicated that adding simvastatin to lipid nanoparticles did not cause any changes to skin biophysical parameters.

***Conclusion:*** The preparation method of simNLC developed in this study is a suitable method, and the nanoparticles fabricated were safe with acceptable long-term stability and drug entrapment.

## Introduction

Vitiligo is a long-term autoimmune problem in which growing patches of skin lose their color on parts of the body. It can affect people of any age, gender, or ethnic group, but causes much trouble among dark skin individuals.^1^ These patches come into sight when melanocytes (melanin-producing cells) within the skin die off. The exact cause of vitiligo is unknown, but it may be due to an autoimmune disorder or a virus.^2^ The estimated prevalence of this disease is 0.5%-1% of the population. The onset of the lesions in 25% of patients is noted before the age of 10, in 50% of them before the age of 20 and in 95% before the age of 40.^3^

There are two types of vitiligo: segmental and non-segmental. Non-segmental vitiligo is more common and is defined as when the lesions are on both sides of the body. Furthermore, in segmental vitiligo, the depigmentation areas are just on one side. Pathology of the disease is unclear, but there are some hypotheses indicating action mechanisms such as autoimmune theory, melanocytorrhagy, oxidative stress, biochemical, intrinsic defect, and neural mechanism that can affect the melanocytes.^4^

Despite recent advances in the treatment of vitiligo, there is still no FDA-approved treatment to inhibit its progression.^5^

One theory blames cluster of differentiation 8 (CD8+) cytotoxic lymphocytes. CD8+ T cells produce interferon gamma (IFNγ), which is a cytokine destroying the melanocytes.^6^ Recent work analyzing IFNγ signaling suggests that ligation of IFNγ receptors leads to activation of signal transducer and activator of transcription 1 (STAT1) and eventually transcription of IFNγ-induced gene.^5,6^ A recent study showed cluster of differentiation 4 (CD4+) T cell played an important role in vitiligo pathogenesis. Cytokines secreted by T Helper cell type 1(Th1) (TNF-a) and T helper cell type 17 (Th17) (interleukin-17 (IL-17), interleukin-22 (IL-22)) are responsible in duration and maintenance of the disease.^7^

Simvastatin is a white, crystalline and poorly water-soluble powder.^8^ It has beneficial effects on hypercholesterolemia.^6,9^ It acts by inhibiting the 3-hydroxy-3-methylglutaryl coenzyme-A (HMG Co-A) reductase.^10^ This enzyme catalyzes the biosynthesis of cholesterol.^8^ The immunosuppressive effect of statins was first observed in 1990 when Pravastatin was reported to reduce heart transplant rejection.^11^

Simvastatin mechanism of action is T cell inactivation by exclusively direct inhibition of IFNγ signaling. Thus, it has a good immunosuppressive action in some diseases like rheumatoid arthritis.^12^ There is some evidence that it can be used as an immune-modulator in preventing and reversing the depigmentation of vitiligo lesions in mouse model owing to the inhibitory effects of simvastatin on STAT1.^5^ Therefore, there have been suggestions that it can be used as a treatment in vitiligo patients. NLCs are new effective drug-delivery systems and have been introduced as an alternative carrier to such other colloidal systems as liposomes, polymeric nanoparticles and nanoemulsions.^13^ The use of NLC is a prominent advance, since the solid matrix of the lipids presents high flexibility in controlling the drug release, protects the encapsulated drugs from degradation, enhances the drug penetration into the skin, increases the loading capacity of active compounds, and minimizes the expulsion of active ingredients during storage.^14-17^ NLCs are usually composed of biodegradable and biocompatible lipids as core coated with safe surfactants as the external shell.^18^ The aim of this study is to fabricate and assess the physico-chemical characteristics and the safety of simNLCs to use them topically on vitiligo lesions.

## Materials and Methods

Simvastatin was obtained as a gift sample from Artemis Biotech (Hyderabad, India), Tween 80 (polysorbate 80) and Cetyl palmitate were purchased from Oleon (Ertvelde, Belgium), Miglyol (Caprylic/Capric triglyceride) and Tego care 450 (polyglyceryl-3 methylglucose distearate) were procured from Evonik Goldschmidt GmbH (Steinau an der Straße, Germany). Propylparaben and methylparaben were obtained from Alborz Bulk (Saveh, Iran). Deionized water was prepared freshly when required.

### Preparation of simNLC 

Briefly, the 12% w/w lipid phase (Tego care 450 (lipid base emulsifier), Miglyol and Cetyl palmitate) and the 85.5% w/w aqueous phase (Parabens, Distilled Water) were heated separately to 75°C. Afterward, simvastatin powder was dispersed in Tween 80 as an aqueous phase surfactant and added to the lipid phase, which was then added to the aqueous phase with continuous stirring using a homogenizer (Ultra-Turrax^®^ T25 IKA Labortechnik, Staufen, Germany), at 8000 rpm for 90 seconds.

The above mixture was subjected to probe sonication (Hielscher ultrasound technology UP400s, Germany) at 70% amplitude for 1, 3 and 5 minutes, separately. The obtained solution was refrigerated at 2–3°C for 15 minutes.

### Characterization of simNLC

#### Physicochemical assessment

The prepared simNLC emulsion was checked to control its pH, color, odor, texture and uniformity.

### Dynamic light scattering (DLS) measurements 

Particle size, polydispersity index (PDI) and zeta potential (ZP) were measured by dynamic light scattering using the Zetasizer (Malvern Zetasizer Zen3600, UK). The NLC formulation was diluted (1:20) with deionized water to obtain the proper scattering intensity, and measured at 90° scattering angle and at a temperature of 25°C.^19^

### Transmission electron microscopy (TEM)

TEM was used to study the morphological features of NLCs. A Philips CM120 TEM (the Netherlands) with 200 KV accelerating voltage was used. A thin layer of the sample was placed on the carbon-coated copper grid for 3 to 5 minutes, and the excess fluid was removed with filter paper and left to air-dry. Then, micrographs from the sample were recorded.

### Powder X-ray diffraction (P-XRD)

P-XRD analysis was conducted to verify the formation of new solid-state.^20^ P-XRD analysis was conducted for unprocessed simvastatin physical mixtures, NLC without simvastatin (placebo) and simSLN, using an X-ray diffractometer X’Pert PRO MPD (PANalytical, Holland). Cu K𝛼 radiation in the scanning range of 2𝜃 = 5՞–80՞ was used with tube current of 30 mA, operated voltage of 40 kV, step time of 20 seconds and step size of 0.02.

### Entrapment efficiency (EE) and drug loading (DL)

Two important parameters among various drug delivery systems are EE and DL.^21-23^ They show how much drug is loaded in nanoparticles.SimNLCs were kept at -20°C for 24 hours and then lyophilized for 48 hours using a Martin Christ Lab freeze-dryer (Germany). 10mg of lyophilized simNLC was accurately weighed and dispersed in 10 mL of Ethanol. The dispersion was centrifuged at 20,000 rpm for 30 minutes. The absorbance of the supernatant was measured using a UV-VIS spectrophotometer (3K30, Sigma, Osterode am Harz, Germany) at 237.5 nm, and finally mass of the drug was calculated with the standard calibration curve. The drug entrapment efficiency and drug loading of the formulation were calculated by the following equations.

$$\%EF=\frac{M\;initial\;sim-M\;free\;sim}{M\;initial\;sim}\times 100$$

$$\%DL=\frac{W_{DL}}{W_{NP}}\times 100$$

Where *“M initial sim* ” is mass of initial simvastatin, *“M free sim”* is mass of free simvastatin present in the supernatant, *“M lipid”* is mass of total lipid, “*W*_DL_” is weight of drug loaded in nanoparticles and “*W*_NP_” is weight of nanoparticles (lipids).

### Differential scanning calorimetry (DSC)

DSC is a thermoanalytical technique used to study the melting and recrystallization behavior of the samples. DSC analysis of samples was conducted using Mettler Toledo DSC823 (Mettler Toledo, Greifensee, Switzerland) at a heating rate of 5°K/min from 25°C to 200°C under the nitrogen flow of 80 mL/min. Samples including 1 mg of lyophilized NLC without simvastatin (placebo), lyophilized simNLC, simvastatin powder, Tego care 450, cetyl palmitate as the lipid phase in the formulation and physical mixture of substances were put into 40 μL aluminum sample pan and immediately sealed with a lid to prevent sample degradation. An empty aluminum pan was used as the reference. After all the measurements, the samples were cooled to 0°C.

### In vitro and kinetic drug release study

The in vitro drug release studies were conducted using static Franz diffusion cell comprising 2 (the donor and the receptor) compartments. The donor compartment consists of 2 open ends where one end is shielded with the cellulose nitrate membrane (0.45 micron, D9725 Sigma, USA) soaked in distilled water for 24 hours before the experiment to fix the pore size during the release test. Approximately 0.5 g of fresh simNLC and free simvastatin were put on the membrane separately and devoted to each donor compartment. The receptor compartment (ethanol in water (70% W/W)) contained a small magnetic bead rotated at a constant speed, and the temperature was maintained at 37°C. 3 mL of the receptor phase were taken at the scheduled time intervals (30, 60, 120, 180, 240, 300 and 360 minutes) and substituted in the donor compartment with fresh buffer to maintain sink condition. The absorbance of the sample was determined at 237.5 nm by the UV spectroscopy. The release data were investigated using several kinetics models including zero order, first order, and Higuchi kinetics. The kinetic release models for the formulation was evaluated and the coefficient of determination (*R2*) for each model was determined based on the data attained during *in vitro* release study.

### Stability study

Long-term stability studies were conducted according to ICH guidelines (at 25±2°C/60 ± 5% RH) for 24 months. Physical and chemical properties, including appearance, aggregation of particles, particle size, PDI, and ZP were determined at 0, 3, 6, 9, 12 and 24 months intervals.^21^

### Safety assessment

Fifteen healthy volunteers consisting of 9 females and 6 males (25-55 years old) were eligible for admission. The randomized, double-blind and placebo-controlled pilot study was conducted in compliance with the ethical principles of the Declaration of Helsinki and the Ethics Committee of Tehran University of Medical Sciences (Acceptance No: IR.TUMS.REC.1394.1007).

After obtaining written consent, simNLC and its placebo NLC were applied to the volunteers’ volar forearms twice a day (via Repeat Open Application test), then the skin biophysical parameters (hydration, transepidermal water loss (TEWL(, pH, melanin and erythema index) were analyzed on the test and control sites, on day 0 (prior to application) and 10 days after application of both formulas.

Volar forearm skin properties before and after application were measured by the specific probes of Cutometer^®^MPA 580 instrument (Courage & Khazaka electronic GmbH, Germany). The erythema and melanin content were calculated by Mexameter MX 18 from the strength of the absorbed and the reflected light. The measurement of TEWL by TEWAmeter TM 300 is based on the diffusion in an open chamber. The pH value and hydration were recorded using a pH meter and corneometer, respectively. Side effects were evaluated by questionnaires and physician visual assessments.

During measurement, the room temperature was set to 20 ± 1°C under constant humidity (35 ± 5%). Before the measurements, volunteers stayed in the test room for 15 minutes for adaptation of skin to room temperature and humidity.

The obtained data were entered in the SPSS software version 20, and paired t-test was utilized to determine the difference of the mean of outcomes between tests and control sides, as well as between the mean outcomes of the test side on days 0 and 10 days after application. The level of statistical significance was set at 0.05.

## Results and Discussion

The mean particle size, PDI, and ZP were determined using zeta sizer. As Table 1 shows, the mean particle size of the simNLC was found to be in the range of 203.7 ± 21.54 to 345.4 ± 9.77 nm. The ZP of the formulation was found to vary between −43.3 ± 5.14 and -49.7 ± 7.778 mV. The PDI of the prepared simNLCs was between 0.221±0.054 and 0.294 ± 0.021. NLCs sonicated for 5 minutes had a smaller size, and better PDI and ZP. Sonication for 0.6 and 1 second cycle had no noticeable difference; therefore, the 0.6-second cycle was chosen to save energy and prevent overheating, aggregation and false DLS readings. Finally, based on particle size and ZP values, the A5 preparation with 0.6-second cycles and 5 minutes of sonication was chosen as the best one.

**Table 1:** Comparison of particle size, ZP and PDI of formula with 0.6 and 1 second cycled sonication for 1, 3 and 5 minutes

**Name**	**Power (W)**	**Cycle**	**Time (s)**	**Particle Size (nm)**	**ZP (mV)**	**PDI**
A1	70	0.6	1	345.4±9.77	-43.3±5.14	0.272±0.03
A3	70	0.6	3	248.9±11.68	-48.7±9.09	0.294±0.021
A5	70	0.6	5	217.2±0.707	-49.7±7.778	0.279±0.004
B	70	1	5	203.7±21.54	-43.4±7.57	0.221±0.054

W: Watt, S: Second, nm: nanometer, mV: millivolt, PDI: Polydispersity index

The formulated NLC exhibited suitable organoleptic characteristics and stability. The particles were found to be white in color and odorless and possessed uniformity.

**Figure 1:**
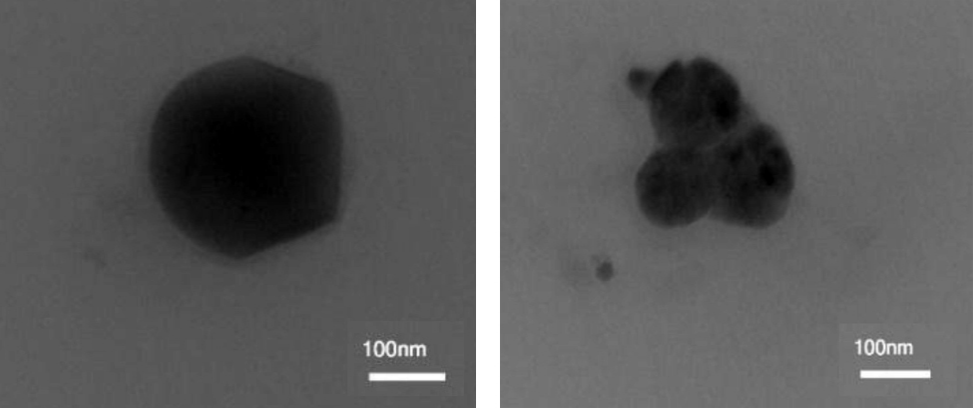
TEM micrograph of simNLC.

Shape and surface morphology of simNLC particles were studied by TEM (Figure 1). TEM analysis indicated solid and spherical-like particles in the nanoscale range with well-defined boundaries.

**Figure 2:**
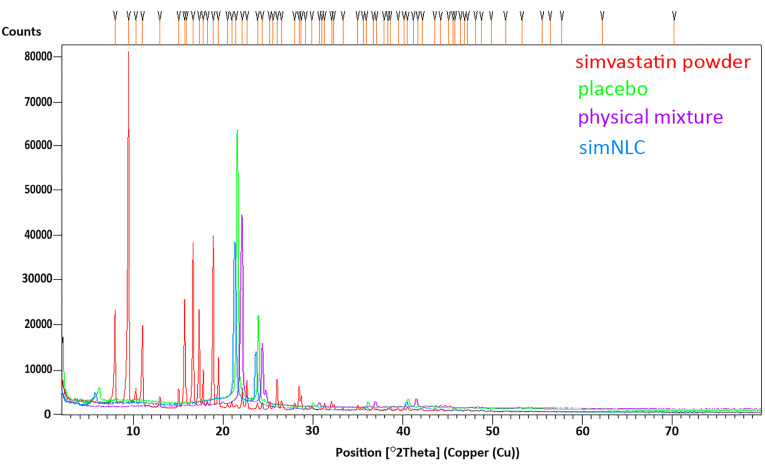
P-XRD curve of physical mixture, placebo, simNLC, and simvastatin powder.

As for P-XRD results, unprocessed simvastatin showed a series of sharp peaks confirming its crystalline nature. However, in simNLCs, most of these peaks were reduced and few disappeared, demonstrating the amorphous nature of the particles (Figure 2). The EE of simNLC and DL was 99.27% and 3.4%, respectively.

**Figure 3:**
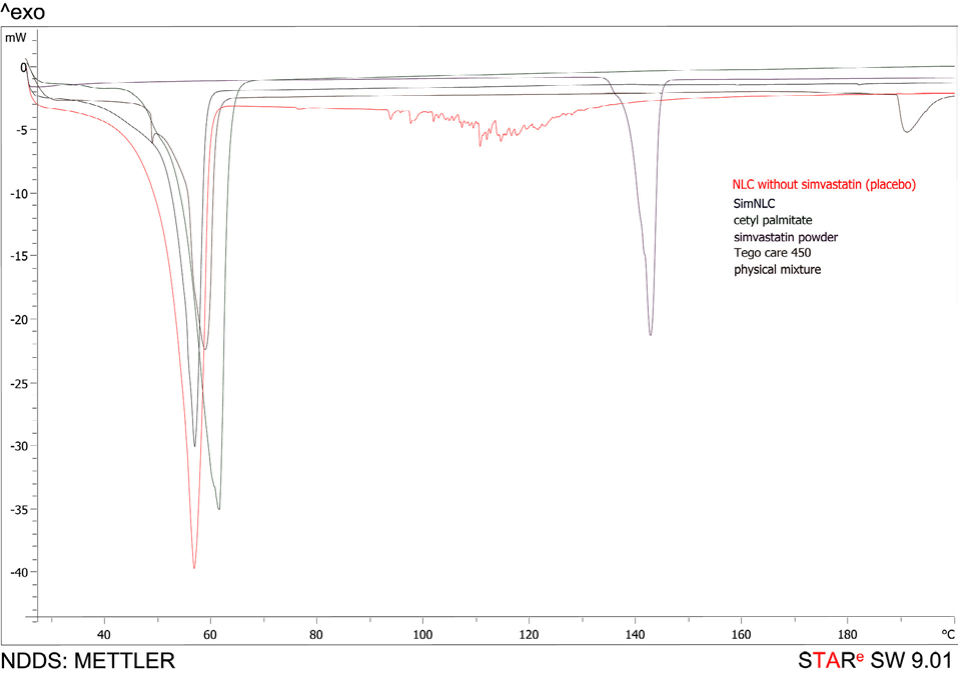
DSC graphs of Tego care450; simvastatin powder; Cetyl palmitate; simNLC; NLC without simvastatin (placebo), and Physical mixture.

DSC thermograms of simvastatin, Tego care 450, cetyl palmitate, lyophilized simNLC, lyophilized NLC without simvastatin and physical mixture were recorded. As Figure 3 shows, their melting behavior changed when the drug was incorporated into NLCs. An endothermic peak was detected for Tego care 450 at 58.16 °C, for cetyl palmitate at 60.30°C, for pure simvastatin at 141.76°C, for NLC without simvastatin at 55.42°C, for simNLC at 55.91°C and for physical mixture at 55.75°C.

**Figure 4:**
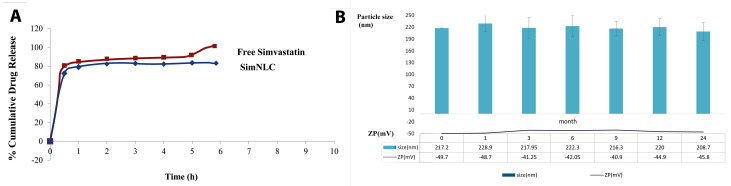
A) The cumulative release pattern of simvastatin from simNLC and free simvastatin during 6 hours and B) Stability study of simNLC in 25±2°C, 60±5 %RH during 24 months.

In vitro release of simvastatin during 6 hours was evaluated by Franz diffusion cell, and the profile is shown in Figure 4A. Cumulative drug release from simNLC at 0.5 and 1 hour depicted a slight slow pattern with lower quantity as compared with free simvastatin and in continue this gap was increased. Based on our outcomes, the release data were kinetically best fitted with the zero order model. The coefficient of determination (*R*^2^) of the obtained equations in the zero order model was near to 1 (Table 2).

**Table 2:** The kinetic mathematical models used to fit the release data

**Kinetic models**	**Zero order** ***(R2)***	**First order** ***(R2)***	**Higuchi** ***(R2)***
Equation	F = k_0_ t	Ln(1 − F) = −k_f_ t	F = k_H_ t ^1/2^
simNLC	0.7841	0.6242	0.771

Parameters of models were obtained by linear regression *(R2)*. ‘F’ represents a fraction of drug released up to time *t*. The *k*_0_, *k*_f_*, k*_H_ are constant of the mathematical models. R2: Coefficient of determination; simNLC: NLC loaded simvastatin.

The stability of simNLC was evaluated for 24 months at 25 ± 2°C/60 ± 5% RH (Figure 4B). These parameters (size, PDI, and ZP) were considered at the mentioned intervals and were found to be relatively constant during the study period. In other words, there was no significant change in particle size, PDI and ZP parameters during 24-month evaluations.

Studies involving comparison of topical application of simvastatin 0.4% in NLC (simNLC) to placebo did not show any significant change in skin biophysical parameters, including TEWL, skin hydration, and hemoglobin content (Erythema), melanin index and skin pH 10 days after application (Table 3).

**Table 3:** Change in skin biophysical parameters after 10 days application of simNLC and placebo

**Parameter**	**Change (%) (mean ±SD)**		***P*** **value**
TEWL	Control	0.0704±0.40933	0.376
	simNLC	-0.0095 ±0.37132	
Hydration	Control	0.0595 ±0.19125	0.797
	simNLC	0.420±0.18442	
Erythema	Control	0.0441 ±0.15391	0.227
	simNLC	0.1026 ±0.10960	
Melanin	Control	-0.1875 ±0.22206	0.538
	simNLC	-0.1481 ±0.15527	
pH	Control	-0.0162 ±0.11778	0.162
	simNLC	0.0441 ±0.13199	

TEWL: Trans epidermal water loss, SD: standard deviation, simNLC: NLC loaded simvastatin.

Formation of nanoparticles is often effective for increasing the bioavailability of active pharmaceutical compounds with poor water solubility.^17^ Therefore, it seems that this kind of drug delivery system can be useful for loading simvastatin to treat the vitiligo disorder.

Some methods for producing NLC include high-pressure homogenization^21^ and ultra-sonication techniques.^22^ Among the methods mentioned, ultra-sonication takes the least time. It is done in 5 minutes compared to hours for other methods.^23^

To prepare NLC, the lipid phase was added to the aqueous phase followed by high shear homogenization to produce homogeneous and white pre-emulsion, then it was sonicated with 0.6 and 1 second cycles and 70% amplitude for 1, 3 and 5 minutes, separately. After that, the formulation was cooled to stabilize and prevent an increase in nanoparticle size.

Particle size and PDI are important characteristics and effective parameters in quality and stability of NLCs. These factors are chiefly influenced by particle structure and production method.^24^ In simNLC, PDI value around 0.2 and ZP of -49.7 indicate that the nanodispersion is stable in nature. Both of these values for simNLC were within the range (PDI <0.3 and |ZP| > 25), exhibited electrostatic stabilization having no aggregation, and prevented Ostwald ripening and particle growth. An electric charge on each particle surface forms electrical barrier which results in ‘Repulsion phenomenon’, which is called the ZP. ZP was found to be toward negative side of −49.7 mV. It supports the fact that the surfactants and the drug used in the formulation of simNLCs possesses negative charge.

EE is defined as the fraction of the drug loaded, which has direct effect on drug release. Moreover, DL is determined as the ratio of the weight of drug loaded to the weight of carriers.^25^ EE and DL are dependent on the active compound, nanoparticles properties, and nanoparticle synthesis method.

Our results confirmed that the almost complete EE showed high efficiency of NLC in taking up the drug simvastatin. The great DL determined approximately complete loading of simvastatin into NLC.

In the TEM image (Figure 1 left side), lack of symmetry at the edges of NLCs was seen. This could be due to the probe sonicator technique, which emits high frequency sound waves and can cause irregularities on the particle surfaces. Moreover, the observed nanoscale size and homogeneous distribution of the nanoparticles were in agreement with those determined in DLS studies.

The results of DSC thermograms demonstrate the absence of the crystalline form of the drug in the NLCs due to the molecular dispersion of the drug in the lipid matrix. The sharp peak of simvastatin at 141.76°C was not seen in simNLC thermogram, suggesting that most of the simvastatin was incorporated into NLC according to the high EE of active powder to simNLC (Figure 3).

In addition, this small diffused peak of simNLC indicated reduced particle size of simvastatin, enlarged surface area, and close contact between solid lipid and drug, confirming the XRD results indicating change of simvastatin from the crystalline to amorphous state.

It has been reported that in polymer and lipid-based nanoparticulate drug delivery systems, the binding energy of the active ingredient with the lipids plays a crucial role in effective encapsulation.^26^ Surfactants play an important role in this regard. Our particles contain mixture of Tween 80 and Tego care 450, with high HLB values, as surfactant. This mixture was suitable for efficient dispersion of cetyl palmitate and simvastatin in the aqueous phase and provided a protective rigid barrier to obtaining appropriate nanoparticles.^27^

Furthermore, the surfactant mixture was appropriate for long-term storage since at room temperature, the formulation remained unchanged for the entire period of the stability test and there was no significant change in color, EE, size, and PDI of the sample (some data were not shown). Thus, it can be concluded that selected materials and the sonication technique led to NLCs, which were stable for 2 years. The good stability and nanoaggregated pattern are owing to the high ZP values of simNLC (|ZP|> 25), resulting in strong inter-particular repulsive forces.

This drug delivery system with acceptable physicochemical properties can release simvastatin within 3 hours. Thus, a complete and appropriate delivery of simvastatin does not influence human skin. No skin abnormalities such as burning, itching and scaling were detected in the volunteers. Additionally, objective investigations of skin biophysical parameters obviously indicated that application of NLC did not destruct the epidermal barrier. The pH of simNLC was found to be in the range 5.5-6.0, which is within the pH of normal skin.^28^ This is the third study demonstrating that the lipidic nanostructures such as nanoemulsions (NE), solid lipid nanoparticles and NLC are safe and tolerable novel drug delivery systems.^29,30^

## Conclusion

We fabricated simNLC by a simple and reproducible technique (ultra-sonication), which was possible to be scaled up for commercial production. Our physicochemical analysis confirmed that simvastatin in the form of NLCs was a boosting nanomedicine to enhance bioavailability with high physical stability and appropriate release profile. Safety study results indicated that the simNLC was a safe carrier to be used as a novel therapeutic treatment in vitiligo disease.

## Ethical Issues

The study was conducted in compliance with the ethical principles of the Declaration of Helsinki and the Ethics Committee of Tehran University of Medical Sciences (Acceptance No: IR.TUMS.REC.1394.1007).

## Conflict of Interest

Authors declare no conflict of interest in this study.

## Acknowledgments

This study was supported by research grant number 94-03-34-30080 from Center for Research and Training in Skin Diseases and Leprosy, Tehran University of Medical Sciences.
